# Mechanical Behavior of AZ31B Mg Alloy Sheets under Monotonic and Cyclic Loadings at Room and Moderately Elevated Temperatures

**DOI:** 10.3390/ma7021271

**Published:** 2014-02-18

**Authors:** Ngoc-Trung Nguyen, Oh Suk Seo, Chung An Lee, Myoung-Gyu Lee, Ji-hoon Kim, Heon Young Kim

**Affiliations:** 1 Graduate Institute of Ferrous Technology, Pohang University of Science and Technology, San 31 Hyoja-dong, Nam-gu, Pohang, Gyeongbuk 790-784, Korea; E-Mail: ntnguyen@postech.ac.kr; 2 Department of Mechanical and Biomedical Engineering, Kangwon National University, 192-1, Hyoja2-dong, Chuncheon, Gangwon-do 200-701, Korea; E-Mails: schoolsos@kangwon.ac.kr (O.S.S); josm1212@kangwon.ac.kr (C.A.L.); 3 School of Mechanical Engineering, Pusan National University Jangjeon-dong, Geumjeong-gu, Busan 609-735, Korea; E-Mail: jhfkim@gmail.com

**Keywords:** yielding asymmetry, anisotropy, hardening, tension/compression loading, Young’s modulus

## Abstract

Large-strain monotonic and cyclic loading tests of AZ31B magnesium alloy sheets were performed with a newly developed testing system, at different temperatures, ranging from room temperature to 250 °C. Behaviors showing significant twinning during initial in-plane compression and untwinning in subsequent tension at and slightly above room temperature were recorded. Strong yielding asymmetry and nonlinear hardening behavior were also revealed. Considerable Bauschinger effects, transient behavior, and variable permanent softening responses were observed near room temperature, but these were reduced and almost disappeared as the temperature increased. Different stress–strain responses were inherent to the activation of twinning at lower temperatures and non-basal slip systems at elevated temperatures. A critical temperature was identified to account for the transition between the twinning-dominant and slip-dominant deformation mechanisms. Accordingly, below the transition point, stress–strain curves of cyclic loading tests exhibited concave-up shapes for compression or compression following tension, and an unusual S-shape for tension following compression. This unusual shape disappeared when the temperature was above the transition point. Shrinkage of the elastic range and variation in Young’s modulus due to plastic strain deformation during stress reversals were also observed. The texture-induced anisotropy of both the elastic and plastic behaviors was characterized experimentally.

## 1. Introduction

Lightweight materials have become increasingly important for the automotive industry with the increase in fuel prices, legislative requirements, and directives for controlling carbon dioxide (CO_2_) emissions. Interest in making cars lighter and more fuel-efficient has encouraged the automotive industry to replace steel, iron, and in some cases, aluminum parts, with magnesium (Mg) alloys due to their unique combination of being lightweight, having high specific strength and stiffness, and being highly recyclable. In addition, Mg alloys have been used for computer, communication, and consumer electronic applications due to their good mechanical properties, resistance to corrosion and aging, and electromagnetic shielding capability.

Warm or hot press-forming technology for Mg alloy sheets has been recently recognized as a promising alternative for conventional die-casting or squeeze-casting methods [1–4]. Basal slips, with the lowest critical resolved shear stress (CRSS), mainly contribute to the deformation of Mg at RT, even though non-basal slip systems and twinning also contribute to a small extent. Additional slip systems must be activated to successfully form Mg sheet-metal alloys. The forming limit can be considerably increased by activating necessary additional slip systems at elevated temperatures [5–10]. Higher temperatures increase the ductility of Mg, which then shows more extensive prismatic slip [1]. In addition, Mg alloy sheets have significantly greater formability at elevated temperatures than at lower temperatures in various sheet-forming processes [10–13], and the formability of wrought Mg alloys can be appreciably improved by suitable local heating and cooling processes [14]. Therefore, in the design and forming conditions of tools including selection of the processing temperature, the temperature-dependent behavior of the material should be correctly obtained.

To accurately model the deformation process for Mg alloy sheets, it is important to develop particular testing systems and to conduct relevant experimental tests. A wide range of twinning temperature effects under complex loading conditions must be accounted for to accurately predict deformation during the stamping process. Hence, the objective of the present study was to provide an experimentally comprehensive characterization of the temperature-dependent responses of Mg alloy sheets at a wide range of temperatures (25–250 °C). This characterization included the temperature-dependence of the material’s tensile modulus, ductility, anisotropy, initial yielding, and plastic flow behavior, as well as the identification of the twinning–slip transition temperature. In particular, attention was directed to the unusual hardening behavior of AZ31B sheets under cyclic loading using a newly developed testing machine [15]. Based on our test results, a calibration process can be applied to improve confidence in simulation results using the correct material parameters for existing constitutive models. Furthermore, the material characteristics can be used to develop a phenomenological constitutive model that sufficiently describes the experimental observations of the hardening behavior of AZ31B sheets under monotonic and cyclic loadings at the tested temperatures. Test data in this study may also contribute to establish an accurate analysis model of springback and formability predictions. In these potential applications partially/locally heated strategy can be applied to control the amount of straining and to save energy, thus the mechanical behavior in a broad temperature range is required.

## 2. Experimental Procedure

Several tests of 1-mm-thick AZ31B Mg alloy sheets were carried out at a series of temperatures, ranging from RT (25 °C) to 250 °C. Monotonic tension tests were conducted in an enclosed atmospheric furnace with a universal testing machine (Instron 5582, Norwood, MA, USA), whereas cyclic loading tests were done with a newly developed device with electrical resistance cartridges as the heating sources. Despite the well-known drawbacks of such heating methods (e.g., issues with extensometry, mechanical pass-through, slow heating rates, and limited isothermality at high testing rates), our method proved useful at reasonably low constant strain rates with the use of optical measuring systems for strain monitoring.

### 2.1. Material

Commercial AZ31B Mg alloy sheets from POSCO [16] were used; their chemical compositions are shown in Table 1. The as-received sheets had dimensions of 500 × 400 × 1.0 mm^3^. All specimens were taken from a single lot of material.

### 2.2. Uniaxial Monotonic Loading Tests

**Figure 1.**
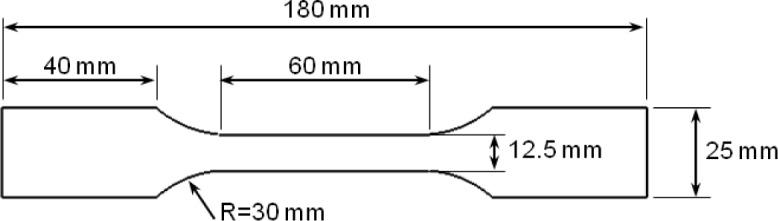
Korean Standard (KS) B0810 13B flat dog-bone specimen.

Korean Standard (KS) B0810 13B flat dog-bone specimens were used for the uniaxial monotonic tension tests. Figure 1 shows the geometry and detailed dimensions of each specimen. The tensile samples were hotwire-machined from one of the alloy sheets at 0°, 45° and 90° from the rolling direction of the sheet. A gauge length of 50 mm was defined using two markers; the actual distance was measured and recorded to calculate the elongation output.

Uniaxial tension tests were performed on an Instron 5582 universal testing machine (Norwood, MA, USA) with a capacity of 100 kN and a maximum crosshead speed of 500 mm/min. The sample temperature was controlled in the heating furnace with a convection heating system mounted on the testing machine. An Instron advanced video extensometer (AVE) was used to measure the elongation of the specimens; this allowed for easy and accurate measurements.

The uniaxial monotonic tension tests were conducted at RT, 100, 150, 200 and 250 °C with the tensile axes of the AZ31B sheet specimens aligned along the rolling direction (RD), diagonal direction (DD), and transverse direction (TD). A heating time of more than 20 min was required to reach the desired temperature before each test was started. The temperature of the chamber was kept constant during each test, and a constant crosshead speed of 0.05 mm/s, corresponding to a strain rate of 0.001/s, was applied.

### 2.3. Cyclic Loading Test

**Figure 2.**
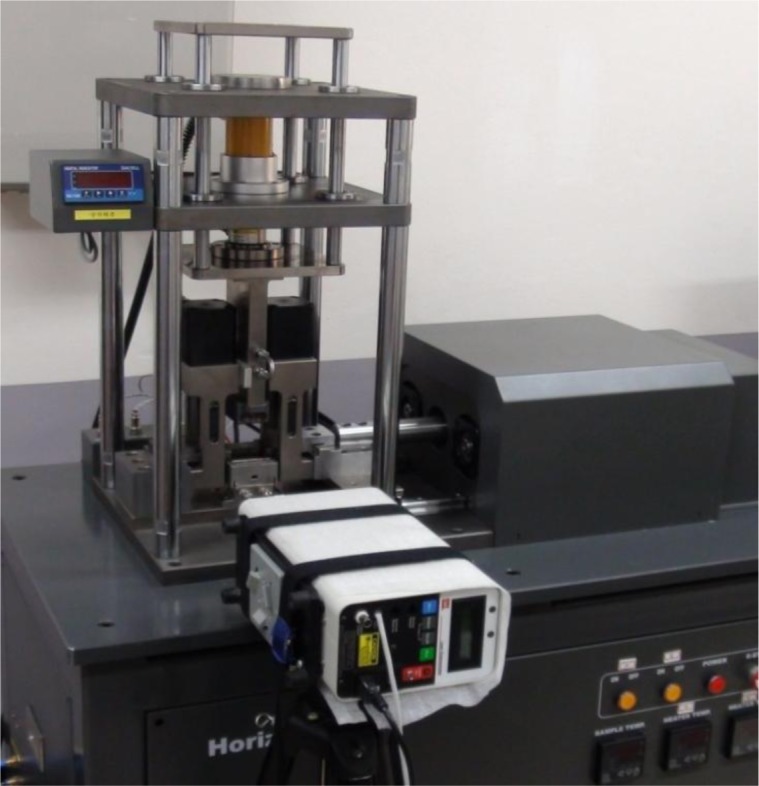
The tension-compression tester of horizontal loading type with upper and lower heating system, anti-buckling system, and laser extensometer to measure axial strain.

A special testing system was required to analyze the main features of the AZ31B Mg alloy sheets at large strains during reverse loading at RT and elevated temperatures. In practice, a compression test of sheet metal at large strain rates is not trivial due to the buckling phenomenon. With its slender geometry, a sheet specimen buckles easily. In this study, a new device was developed, as shown in Figure 2.

A side force was applied to stabilize the compressive deformation, suppressing the buckling of a typical tensile specimen. The loading system of the device was equipped with an alternating current (AC) motor to generate motion in the horizontal direction. The anti-buckling apparatus consisted of comb jigs, allowing a large compressive strain to be applied to the specimen without buckling. Additional constraints in the thickness direction were controlled by the side force applied to the jigs. This force was generated either by a deadweight or a hydraulic loading system. In addition, alignment of the specimens was straightforward, minimizing the production of imperfections in compressive loading, which trigger early buckling. However, friction between the specimens and the jig surfaces was unavoidable. Lubrication was applied between the moving surfaces. There were two heating cartridges embedded in the upper die, and three others in the lower die. The temperature of the tool was monitored during tests with an installed thermocouple. By adjusting the temperature of the cartridges in the upper and lower dies independently, the target temperature in the specimen was effectively controlled via direct conduction through the contact surfaces. However, with the use of the heating and anti-buckling systems, only a limited area of the specimen was exposed, and there were not many options available to measure the axial strain with high accuracy. Therefore, an indirect method of strain measurement was used. A laser extensometer was used to measure the distance between two markers on the side of the specimen. These markers were made by attaching special-purpose tape to the correct positions to define the gauge length of the specimen.

**Figure 3.**
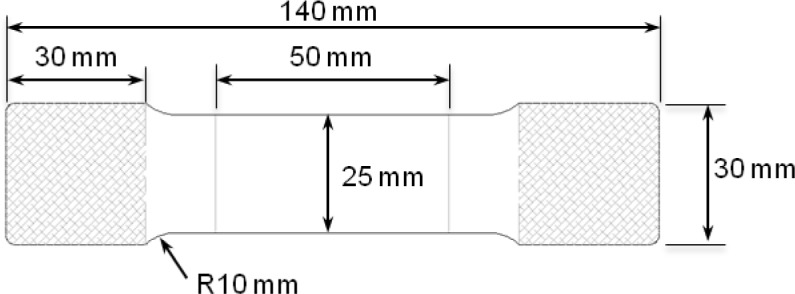
Specimen for cyclic loading test based on ASTM-E8.

Similar to previous studies, e.g., [17,18], an exaggerated dog-bone specimen based on the standard ASTM tensile specimen [19] was optimized by finite element analysis (FEA) simulations to suppress buckling in the thickness direction. Figure 3 shows the specimen geometry, with a rectangular cross-section of 25 mm width and 50 mm length, and a thickness of 1 mm. A gauge length of 50 mm was assigned to monitor the deformation of the specimen during testing. The specimens were hotwire-machined parallel to the RD, TD, and DD.

All cyclic loading tests were carried out using the newly developed tester with a 30-kN load cell, double-side heating system, and a laser extensometer, as shown in Figure 2. Compressive strains of about −8% could be achieved in the cyclic tests, and homogeneous strains were accurately measured using the non-contact laser extensometer. The target temperatures and time-to-temperature were set directly on a computer. The limit strain values in each cycle were also controlled to obtain different pre-strain values. Note that the same nominal strain rate of 10^−3^/s was used, and different optimum values of the deadweight were applied at each temperature. Through these settings, continuous tension–compression–tension (TCT) and compression–tension–compression (CTC) tests were carried out at five different temperatures (25, 100, 150, 200 and 250 °C) to examine the effect of changes in the strain path on the typical stress–strain behavior of the AZ31B Mg sheets.

**Figure 4.**
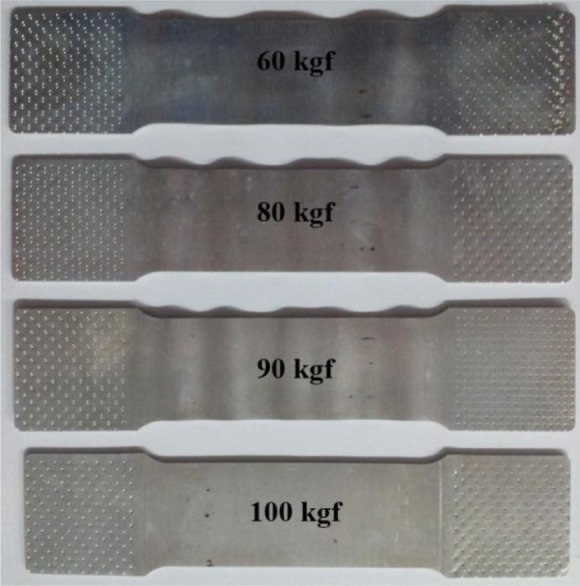
Buckling of specimen at low values of side force.

Cyclic testing using the newly developed system was conducted as follows. First, the specimen was clamped onto the specimen jig. Then the anti-buckling device was attached to the top surface of the specimen from the vertical loading frame, providing the side force through the application of a deadweight. The optimal value of the deadweight was determined from an experimental case study. The case studies showed wrinkles in the specimens with low side forces, as depicted in Figure 4.

A 100-kgf deadweight was selected for this study. For tests at elevated temperatures, a heating step up to the given condition was required before clamping the specimen to avoid unwanted thermal-induced stress. Heat sources from the upper and lower heating systems were maintained during the test to achieve an isothermal testing condition. Next, a tensile force was applied within the prescribed pre-strain, and the loading direction was gradually reversed to compression. This procedure was repeated for the next cycle of tension followed by compression. A similar procedure was followed for CTC loading. For springback prediction, in which the main strain path is tension–compression or compression–tension, just one cycle of the test was sufficient. However, more cycles can be applied if necessary.

Biaxial stress and friction corrections were introduced to obtain the equivalent uniaxial plastic behavior, following a previously described procedure, with some modifications [17,20]. As a side force was applied to the specimen to suppress buckling during compression, the effects of biaxial loading and friction should be corrected. The procedure for this correction can be found elsewhere [17,20]. In all of the calculations, the biaxial effect due to the stress normal to the thickness caused by the side force was negligible. However, the influence of the friction effect could not be neglected, even with the use of different types of lubricant. A series of tests with a split specimen was used to determine the friction force of the testing machine at all investigated temperatures [15]. Calibration of the new testing system was performed using the uniaxial tension test data described in the previous section.

### 2.4. Unloading–Loading Tensile Tests

The inelastic recovery behavior of metals after plastic deformation, *i.e.*, the unloading stress–strain curve, is not linear, and the total strain recovery during unloading is larger than that calculated by the given elastic modulus [21–24]. The inelastic strain released from a plastic deformation state is a major source of additional strain recovery [24]. The elastic modulus of most metals decreases after plastic deformation [25,26]. The diminution of the elastic modulus during plastic deformation can be more than 10% of the initial value after 5% plastic strain [27,28]. Similar trends of decreasing elastic modulus with increasing pre-strain during cyclic tension–compression deformation have also been observed in cyclic tests [27]. As suggested by many authors, the elastic modulus varies with the plastic strain [25–27,29–32].

#### 2.4.1. Unloading–Loading Tension Test

The unloading behavior of AZ31B Mg sheets was characterized using the same experimental setup and specimens that were used for the tensile tests described above. The specimens were loaded to a certain plastic strain level, and then unloaded. After they were fully unloaded (until the force disappeared), the material was reloaded to a higher level of plastic strain, and then again unloaded. This procedure was repeated for several values of plastic strain. Two loading control methods were used simultaneously in this test: the force-control and the displacement-control methods. In the loading phase, the displacement method was applied to control the plastic strain level. However, in the unloading phase, the force method was used to guarantee a fully unloaded condition by the end of the phase. For elevated temperatures, the loading conditions were limited to ensure that the maximum stress value did not exceed the ultimate tensile strength. This was required because a softening effect due to microvoid growth may occur, affecting the Young’s modulus.

#### 2.4.2. Unloading–Loading Compression Test

The procedure used for the tension tests was also applied for the compression loading–unloading–reloading tests, which were conducted using the newly developed testing system. The specimens described for the cyclic tests were also used for these tests. The optimized side force described in the previous section was also applied here to avoid buckling. Similar to the tension loading tests, the specimen was first loaded to a given level of plastic strain, and then unloaded. Then, the specimen was reloaded to a higher level of plastic strain, and again unloaded. This procedure was repeated for several values of plastic strain for all of the investigated temperatures. The number of cycles and the strain levels were restricted to limit the cycle fatigue and fracture effects of the material.

## 3. Results and Discussion

### 3.1. Uniaxial Monotonic Loading Tests

**Figure 5.**
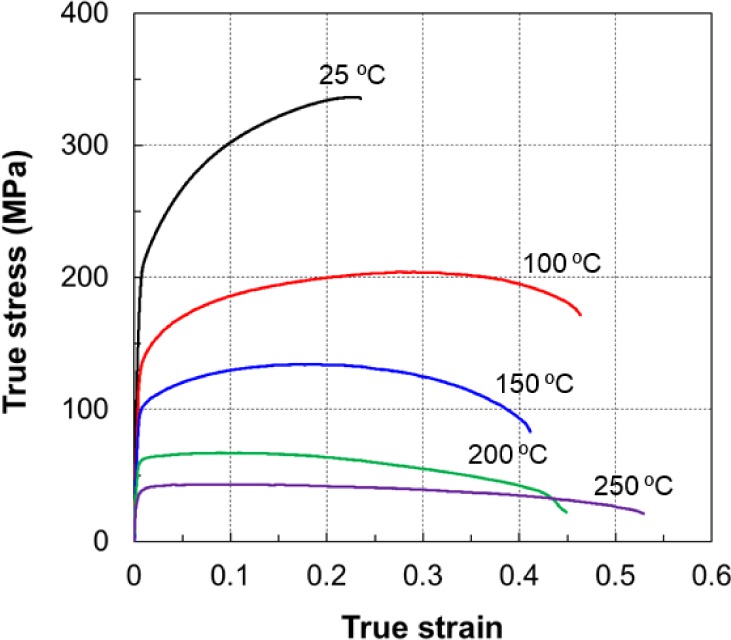
Stress-strain curves in uniaxial tension test at different temperatures.

All uniaxial monotonic tension tests were carried out at a nominal strain rate of 10^−3^/s and a constant crosshead speed of 2 mm/min, and were performed for several elevated temperatures in the range of 25–250 °C, with the results of three tests averaged for each temperature. The results of the tensile tests at various temperatures are shown in Figure 5. Generally, heat generation due to dissipation of plastic work related to high strain-rate testing will affect the temperature of the specimen. However, as the strain rate at which the tests were performed was low, this effect could be ignored and a uniform temperature in the gauge length was assumed.

#### 3.1.1. Effects of Temperature on the Flow Curve

The maximum elongation before fracture at RT was limited to less than 25%. The yield stress dropped significantly as the temperature increased, whereas the elongation improved and reached 55% at 250 °C. As shown in Figure 5, as the temperature increased, the flow stress decreased and the total elongation increased, and the material was clearly softer at temperatures of 200 °C or higher. The hardening rate decreased as the temperature increased, and there was almost no observed hardening effect at 250 °C. This makes it harder to obtain uniform deformation in specimens at elevated temperatures.

**Figure 6.**
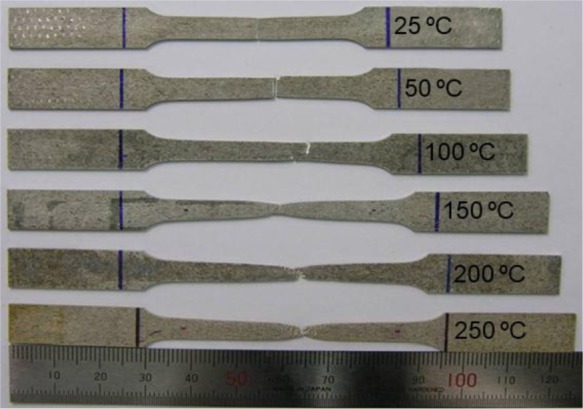
Specimens after uniaxial tension test at different temperatures.

The post-mortem specimens in Figure 6 show abrupt fracture behavior of Mg alloy sheets at and slightly above RT. The brittle nature of fracture for AZ31B, which occurs with little evidence of plastic localization (necking) in either the width or thickness directions, was quite different from the ductile failure due to plastic strain localization for low-strength sheet alloys with cubic crystal structures. Tensile fractures were observed at elevated temperatures, showing significant plastic localization before failure. The generalized ductility was greatly enhanced and the elongation of the specimens increased as the temperature increased.

#### 3.1.2. Anisotropy

It has long been recognized that metals with HCP crystal structure exhibit noticeable anisotropy of physical and mechanical properties [33,34]. The rolling process aligns the {0001} basal plane so that it is parallel to the sheet plane due to the easy slip that occurs on the basal plane along the 〈*a*〉 direction in Mg alloy, resulting in a strong $\{0001\}\langle 11\bar{2}0\rangle$ texture. The pronounced anisotropy in the plastic deformation behavior is attributed to the strong basal plane texture of rolled Mg alloy sheets, which occurs as the basal plane of the HCP lattice is aligned parallel to the sheet plane [35].

**Figure 7.**
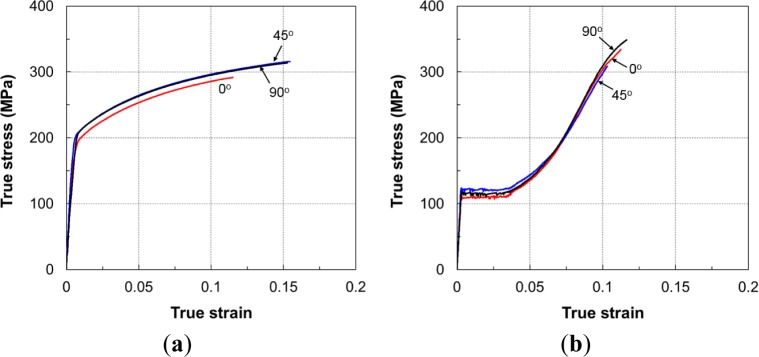
Flow curves of different specimen orientations at room temperature: (**a**) tension loading and (**b**) compression loading.

The same testing procedure used for the uniaxial tension tests was also applied to evaluate the anisotropy of the material. The flow curves at RT are shown in Figure 7.

**Figure 8.**
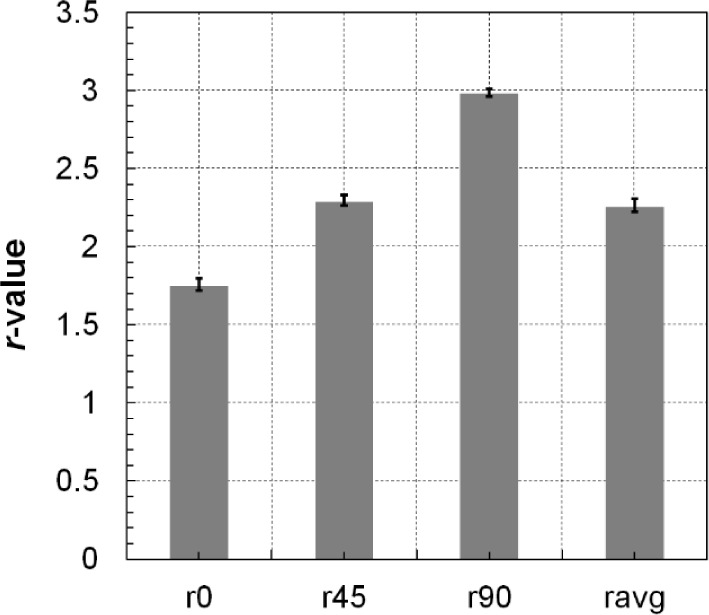
Dependence of the plastic anisotropy parameters on specimen orientation at room temperature.

The curves of specimens at different orientations were only slightly different. The yield point and flow stress in the DD and TD orientations were higher than those in the RD orientation for both tension and compression loading. Figure 8 shows the *r*-values in the RD, DD, and TD at RT. The material exhibited the highest *r*-value of about 3.0 in the TD. This was due to the fact that only a small amount of strain could be accommodated in the thickness direction; therefore, the deformation in the tensile test was mostly induced by the strain in the width direction.

#### 3.1.3. Temperature-Dependent Young’s Modulus

Studies of the temperature-dependent Young’s modulus of Mg alloys are limited [36–39], and different tendencies of the temperature dependency have been reported. Hama *et al.* [37,39] showed that the Young’s modulus is almost constant from RT to 100 °C, and decreases at temperatures greater than 150 °C. They reported values of 42 and 27 GPa at 25 and 225 °C, respectively. In a more recent study on the hot-forming characteristics of AZ31 sheets, Yang *et al.* [36] showed a steady reduction in Young’s modules with a high slope from ambient temperature to about 125 °C. The values remained almost constant in the range of 125–200 °C, and finally decreased at a slower rate when the temperature was higher than 200 °C. They reported values of 40.2 and 27.5 GPa at 20 and 250 °C, respectively. Even though the reported Young’s moduli from these two investigations were only slightly different at ambient and elevated temperatures, the values between these two temperatures were dissimilar in both tendency and magnitude. However, the value of 27 GPa can be used for temperatures above 225 °C.

**Figure 9.**
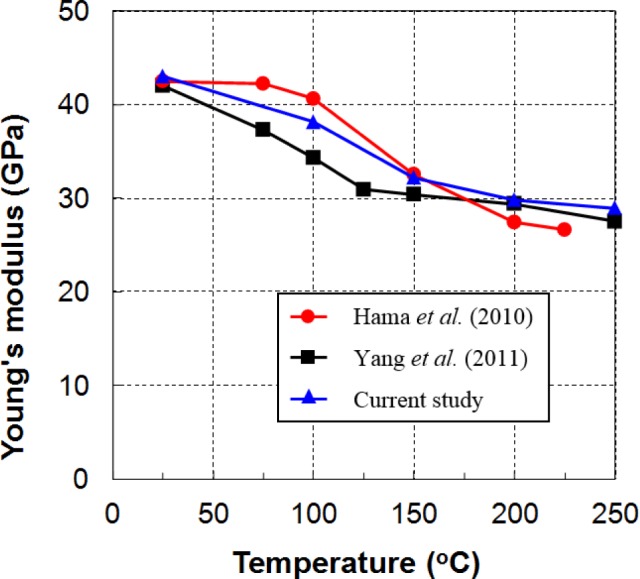
Variation of Young’s modulus with temperature.

Young’s modulus was determined from the slope of the stress–strain curve obtained from the uniaxial tension tests in this study. The tests were performed while taking care to avoid potential slipping at the grips. A more heuristic method for determining this important material constant can be found elsewhere [40]. The measured data and the values from references are plotted on the same graph in Figure 9. Our data are in qualitative agreement with the values provided by Hama *et al.* [37,39].

To estimate the influence of temperature on the amount of springback, the ratio between the initial yield stress and Young’s modulus was calculated at each measured temperature. Values from both the compression and tension initial yield stresses were employed in the calculation. Note that the correct magnitude of the springback should be computed from the residual stress after forming, which is dependent not only on the initial yield stress, but also on the hardening rule and the amount of deformation. Table 2 shows the simplified predictions for the tendency of the springback magnitude with increasing temperature. A drop in the stress-to-stiffness ratio was evident. In particular, the value of this ratio at 250 °C became small, reflecting the vanishing springback observed in our previous tests as the temperature exceeded that level [41].

## 3.1.4. Variation in Young’s Modulus with Increasing Plastic Strain

**Figure 10.**
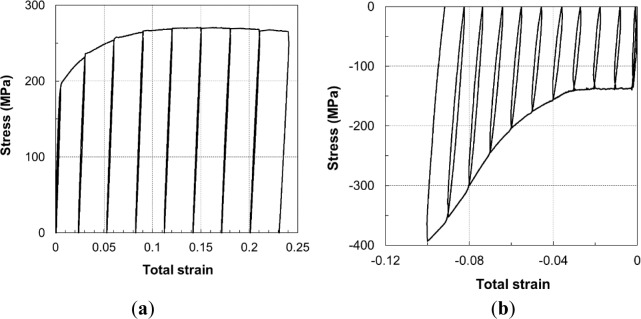
Stress-strain curve in the loading-unloading-reloading tests at RT for: (**a**) tension and (**b**) compression cases.

The typical stress–strain relationships of the unloading–loading tension and compression tests are presented in Figure 10. For different testing temperatures, the range of plastic strain was different to account for the improved ductility as the temperature increased. The strain levels at RT were 1%, 4%, 7%, …, whereas, at 150 °C, they were 1%, 5%, 9%, …

**Figure 11.**
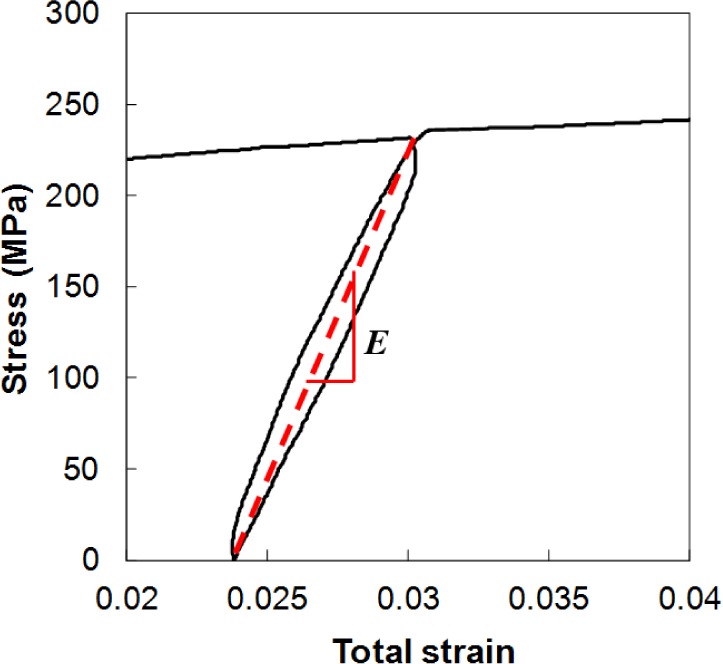
Illustration of the stress-strain relationship in the tension loading-unloading-reloading test at RT.

**Figure 12.**
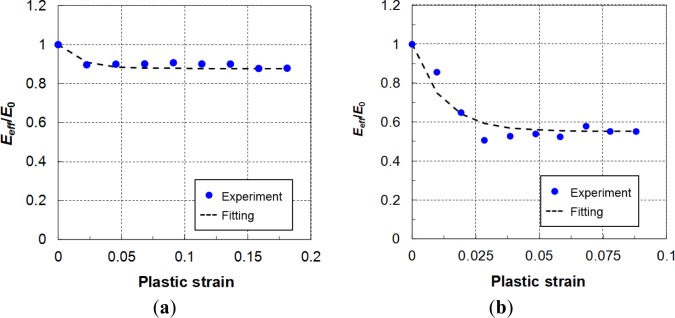
Variation of the ratio between the effective and initial Young’s modulus *E_eff_*/*E*_0_ with plastic strain at RT for: (**a**) tension and (**b**) compression cyclic loadings.

Figure 11 shows that the AZ31B Mg sheets exhibited significant strain hysteresis during unloading and reloading, which is consistent with previous observations in the literature [39]. In this case, the classical assumption of linear elastic unloading behavior with a slope equal to the Young’s modulus of the material after plastic deformation was not valid. A close-up view of the curves showed that the unloading and reloading behavior was nonlinear; the two curves were different. For a cycle, the unloading and reloading curves deviated from the line connecting the unloading point and the zero loading (fully unloaded) point; the line represents the secant to the curves, and its slope defines the effective or mean Young’s modulus, which is generally not equal to the initial Young’s modulus [42,43]. The slope of the secant, or the effective Young’s modulus, was measured during the unloading–loading test. These values were strongly influenced by the amount of plastic strain, as shown in Figure 12 for the case of RT. The relative modulus, which is defined as the ratio between the effective and initial values, decreased when the accumulated plastic strain increased. The measured data exhibited the same tendency as reported in the literature for different materials [27,42–44]. According to Yoshida *et al.* [44], the evolution law of the effective Young’s modulus can be expressed in terms of an exponential relationship as follows:

$$E_{eff}=E_{0}-(E_{0}-E_{a})\left[1-\text{exp}\left(-\xi {\bar{\varepsilon }}^{p}\right)\right]$$ (1)

where *E*_0_ and *E_a_* are the values of the initial and an infinitely large plastic strain of the effective Young’s modulus, respectively; ξ is a material parameter and ${\bar{\varepsilon }}^{P}$ is the effective plastic strain. The initial Young’s modulus was measured as described in the previous subsection. Fitted curves of the relative modulus, *E_eff_/E_0_*, for the two cyclic loading cases are shown together with the experimental data in Figure 12.

The dependency of the effective Young’s modulus on the plastic strain shows different tendencies for tension and compression loading. This is possibly due to the different texture evolution as the material was under tension and compression loading. While a negligible amount of twinning was detected during tension loading, the areal fraction of twinning has been reported to increase rapidly and saturate with increasing compression plastic strain [45,46].

The nonlinear unloading–reloading behavior is explained by the appearance of additional *microplastic* or *anelastic* strains [42,47]. These strains are caused by small-range motions of less stable dislocation structures between obstacles, such as pile-ups. In addition, Hama and Takuda [48] reported that the inelastic response during unloading is related to the activation of only basal slip systems during unloading. According to these authors, the basal slip systems are primarily activated owing to their low strengths compared to the prismatic slip systems. Both prismatic and basal slip systems are initially activated during loading, but the more dominant prismatic slips determine the overall stress level during loading. Upon unloading, the prismatic slip systems are not ready to be activated due to their high CRSS, while the basal slip systems are ready in the opposite direction when their resolved shear stress changes signs because of the inhomogeneity of the material. As a result, only the activated basal slip systems contribute to the inelastic behavior during unloading.

### 3.1.5. Effect of Strain Rate on the Flow Curve at 250 °C

Significant softening responses have been observed in materials as the temperature increases [49]. Likewise, in this study, the material showed an increase in softening as strain rate decreased. However, research on strain softening is still limited [50]. The post-peak portion of the stress–strain curve is normally neglected to simplify the flow stress formula [51,52].

To quantify the effect of the strain rate on the investigated alloy at elevated temperatures, additional uniaxial tension tests were performed for selected temperatures. Strain rates of 0.001, 0.01, 0.1 and 1.0 s^−1^ were chosen by referring to real production processing and machinery for Mg alloy products. The experimental setup was identical to the tests described in the earlier section; the only difference was the control of the constant true strain rate. To achieve constant true strain rates during these additional tests, the crosshead speed was controlled to follow an exponential decay function with time [1]. Processing of the experimental data, *i.e.*, conversion of the force–displacement data into true stress–strain curves, was done using standard equations.

**Figure 13.**
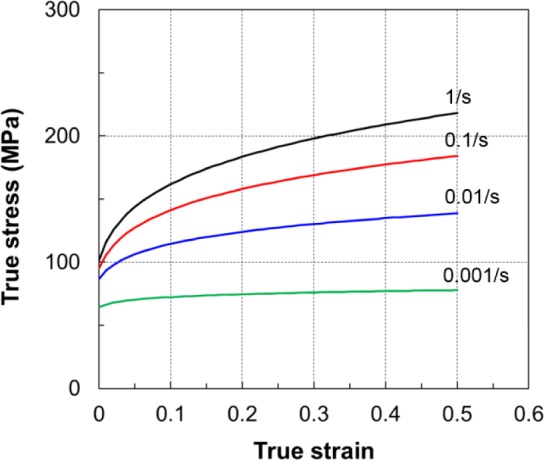
Effect of strain rate on the flow stress at 250 °C (with the specimen of 1.4 mm thickness).

The true stress–strain curves at different strain rates for 250 °C are shown in Figure 13. The flow curve at the lowest strain rate was almost at a steady state. When the strain rate increased, the stress–strain curves shifted upwards and the hardening rate increased significantly.

### 3.2. Cyclic Loading Test

**Figure 14.**
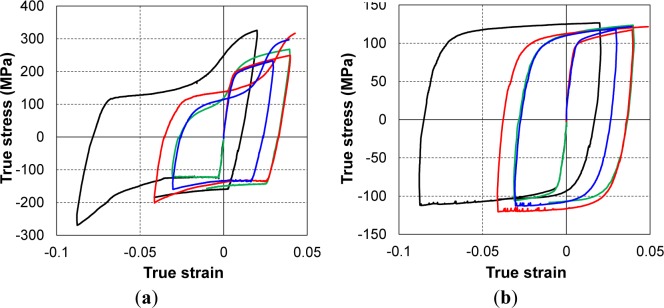
Stress-strain hysteresis loops of cyclic test at: (**a**) room temperature and (**b**) 150 °C.

The typical data shown in Figure 14 reveal the behavior of Mg AZ31B at RT and 150 °C in terms of pre-strain/strain reversals. These results validate the performance of the new testing machine: it could measure a large compressive strain without buckling. Such data are sufficient to develop an enriched one-dimensional constitutive model for corresponding strain reversals [53]. The cyclic behavior of the material at all investigated temperatures was also successfully obtained using the newly developed testing system. The details of some measured characteristics are presented and discussed in this section; the temperature-dependent behavior of the material is emphasized.

#### 3.2.1. Initial Yielding and Flow Stress Asymmetry

##### 3.2.1.1. Initial Yielding Asymmetry

**Figure 15.**
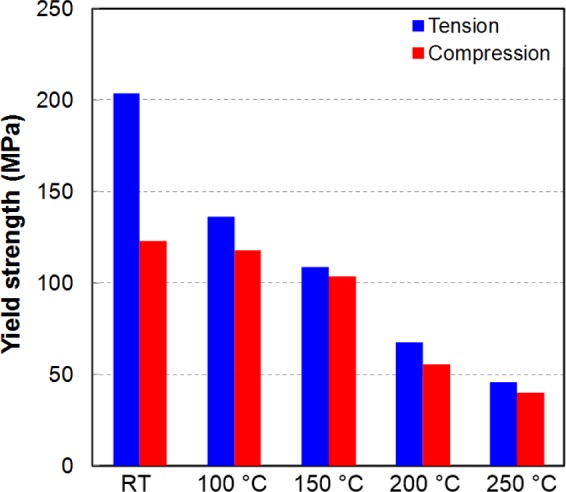
Comparison of yield stress in uniaxial tension and compression tests at different temperatures.

**Figure 16.**
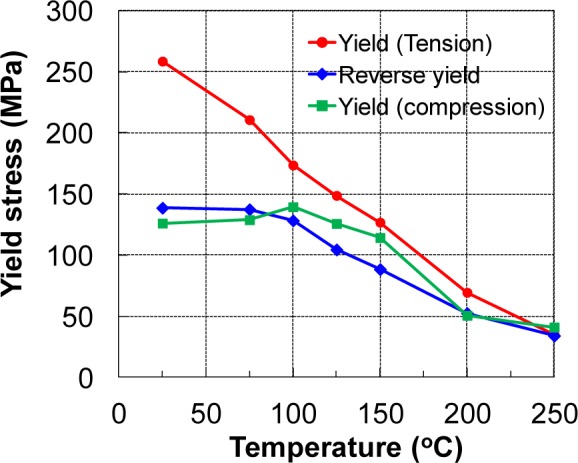
Dependence of yield stress on temperature for the tested cases.

The results of the TCT and CTC tests at five investigated temperatures are shown in Figure 15. The measured stress–strain curves at RT also showed that there was significant asymmetry between the yield stresses in tension and compression. The tensile yield stress was almost double the compressive stress at RT. The difference was reduced significantly with increasing temperature, and almost disappeared at 250 °C. Figure 16 shows the variation in the yield stress with respect to temperature for three loading modes: tension, compression, and compression after tension. The yield stresses during reversal loading after tension were close to the initial yield stress values for compression loading. As discussed below, these two loading cases activated the twinning deformation mechanism, which was relatively temperature insensitive (athermal) when the temperature was less than a critical value; at elevated temperatures, the easily activated slip systems caused the yield stress to be similar for both forward and reversed loading.

##### 3.2.1.2. Flow Stress Asymmetry and Unusual Hardening Behavior

The stress–strain curves of the initial tension loading case at all investigated temperatures showed a general shape similar to that of other metals. The hardening rate decreased as the accumulated plastic strain increased. The flow stress was almost steady at elevated temperatures, and an almost zero hardening rate was observed. However, the stress–strain of compression loading and during tension after compression loading revealed different tendencies. Near RT, the stress–strain curve of compression loading first exhibited elastic behavior, which was followed by an early low-stress plateau corresponding to non-hardening behavior [45,54]. Elastic–plastic transition occurred abruptly and at a much lower stress than the yield point in the tension loading case. After the low stress plateau, the hardening rate started to increase, causing the stress–strain curve to form a concave-up shape. Such behavior is typical of the activation of deformation twinning. No inflection point was recorded within the tested strain levels up to 5%.

**Figure 17.**
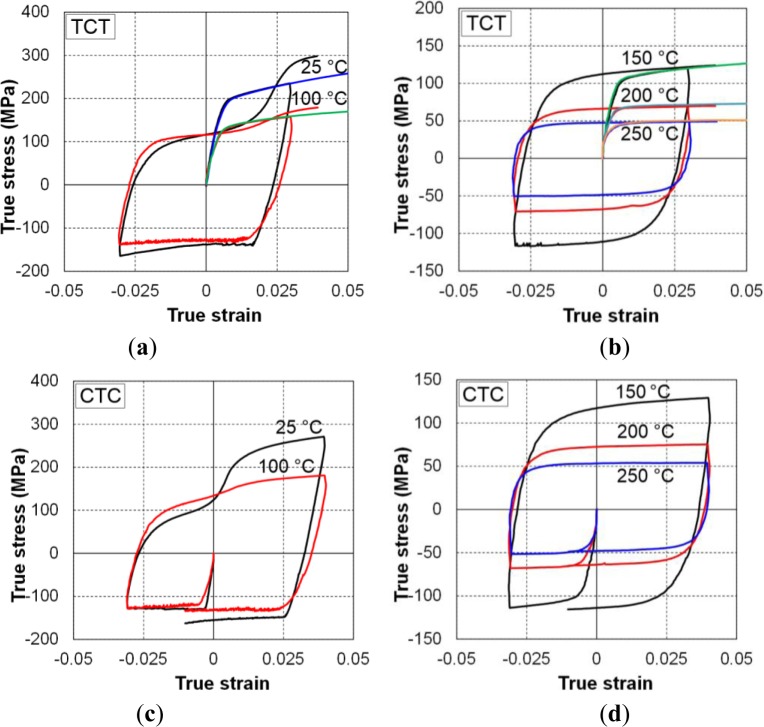
Uniaxial tension-compression-tension (TCT) and compression-tension-compression (CTC) curves at different temperatures: (**a**) TCT at RT and 100 °C; (**b**) TCT at 150, 200 and 250 °C; (**c**) CTC at RT and 100 °C; (**d**) CTC at 150, 200 and 250 °C.

The tension following compression curves also exhibited early yielding at a stress value much lower than that observed at reversal. This is the well-known Bauschinger effect. Unlike the case of compression loading, a smooth transition from the elastic to plastic regions was observed. In the first region, right after the yield indicated by the stress–strain curve, the hardening rate decreased and became zero at a certain strain level. Then it increased rapidly before gradually decreasing again in the second region. This formed a concave-up shape and an inflection point in the flow curve, resulting in a sigmoidal (S-shape). This is typical of an untwinning deformation mechanism. Figure 17 clearly shows the Bauschinger effect, which is defined as early re-yielding upon load reversal. Transient behavior just after the reversed plastic deformation was also observed, *i.e.*, a rapid hardening rate and saturation following re-yielding.

An explanation for the flow stress asymmetry and the formation of S-shaped stress–strain curves follows. For tensile or compressive stress in the rolled axis of the Mg alloy sheet, the most active deformation mode basal slip is not favorable. This can be attributed to the fact that the loading axis is aligned nearly parallel to the basal planes in a strongly textured specimen so as to make the resolved shear stress for basal slip nearly zero. Non-basal dislocation slip occurs under tension [55,56], and such a stress may limit the basal 〈*a*〉 slip but increase the non-basal slip [55,57], which is activated to accommodate deformation when both the basal 〈*a*〉 slip and the most active twinning $\left\{10\bar{1}2\right\}\langle 10\bar{1}1\rangle$ are suppressed when the axis of the tensile stress is parallel to the basal plane. Twinning is the only active deformation mode that can provide straining along the *c*-axis at RT or low temperature [58], even though the mechanism of hard deformation of the dislocation slip with Burgers vectors 〈*c*+*a*〉 has been reported [55,56]. A monotonic compressive curve with twinning typically exhibits a sigmoidal shape, where a low-stress plateau is initially observed, followed by a higher hardening evolution rate.

At temperatures of 150 °C and higher, all of the hardening curves had the “normal” concave-down behavior observed for cubic and non-twinning metals. The existence of a transition temperature was expected to divide the two different hardening behaviors. Switching from one behavior to the other can be explained as competition between twinning and slip-deformation modes, which in turn depend on the temperature, as discussed below. The strong in-plane tension–compression asymmetry near RT, which is reduced at elevated temperatures, can be explained by the activation of mechanical twinning $\left\{10\bar{1}2\right\}\langle 10\bar{1}1\rangle$ during compression along the RD, but not when there is tension along the same direction. This is primarily the result of strong textures, where a significant fraction of the grains are orientated with their *c*-axis nearly perpendicular to the prior working direction RD, and the polar nature of twinning. At ambient temperature, $\left\{10\bar{1}2\right\}$ twinning is the only possible deformation mechanism of elongation in the *c*-axis direction due to difficult activation of other slips [58,59]. Among deformation mechanisms in the *c*-axis direction, $\left\{10\bar{1}2\right\}$ twinning has the lowest CRSS compared to other slips. However, the CRSS for 〈*c*+*a*〉 pyramidal slip decreases with increasing temperature, whereas that of twinning is relatively temperature insensitive [60,61]. Consequently, twinning and 〈*c*+*a*〉 pyramidal slip are competing mechanisms, and they are responsible for deformation in the *c*-axis direction at elevated temperatures. Increasing the temperature facilitates the activation of 〈*c*+*a*〉 pyramidal slip (and possibly other non-basal 〈*a*〉 slips), and hence increases its activity. Presumably, there is a critical temperature at which the CRSS of the two competing deformation mechanisms are balanced. Above this level, deformation by 〈*c*+*a*〉 pyramidal slip is easier, and the flow becomes slip-dominant.

##### 3.2.1.3. Dependence of Yield Stress on Prestrain Values

As reported in the literature, the hardening behavior of Mg sheets was strongly affected by the areal fraction of twinning [45]; twinning deformation can generate new surfaces, which then become barriers to the slip motion of dislocations in the material [62]. However, measurement of the areal fraction of twinning is not trivial [45].

**Figure 18.**
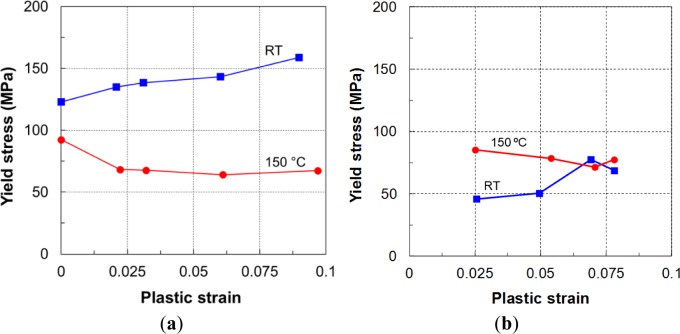
Dependence of yield stress on pre-strains for the isothermal testing cases at room temperature and 150 °C: (**a**) compression loading (twinning deformation mode); and (**b**) tension following compression loading (untwinning deformation mode).

Characterization of the direct dependence of the hardening behavior, particularly the yield stress, on the amount of twinning is not practical. Therefore, the plastic strain was proposed as a measure of twinning in the material caused by prior loading. It has been reported that the increase in strain in compression loading results in an increase in the areal fraction of twinning; a plastic strain level above 8% can achieve twinning saturation [45,46]. The yield stress is thus connected to the accumulated plastic strain according to the prior loading condition, which is implicitly related to the areal fraction of twinning. Such a pre-strain value is accumulated from the most recent yielding until the last unloading point before the reversed loading is applied. Alternatively, a net prestrain defined by the difference of accumulated plastic strains in tension and compression can be used [63]. Relation of the yield stress and the net prestrain is not presented in this paper, interested readers may refer to [63] for a more detailed discussion. In the present study, the yield stress for both compression (twinning deformation mode) and tension following compression (untwinning deformation mode) loadings was recorded and plotted with respect to the pre-strain. Typical curves of the yield stress–pre-strain for the cases at RT and 150 °C are depicted in Figure 18. The yield stress at 150 °C is expected to be lower than that at RT in either loading cases. We speculated that slip deformation mechanism at elevated temperature could be triggered more easily than either twinning or untwinning mechanisms at RT. However, only the yield stress for compression, Figure 18a, shows the case whereas an opposite tendency is observed in Figure 18b for tension following compression. The results in Figure 18b might be interpreted as that untwinning deformation mechanism at RT was activated even with more ease than slip was at elevated temperature.

#### 3.2.2. Twinning–Slip Transition Temperature

The results shown in Figure 16 suggest a method for revealing the transition temperature that distinguishes the two phenomenological deformation modes, based on purely mechanical observations of the compressive stress–strain curves: twinning and slip. For test temperatures up to and including 125 °C, the compression (and tension following compression) curves show a concave-up or inflected appearance characteristic of the activation of deformation twinning, as reported elsewhere [18,45,54]. For test temperatures of 150 °C and higher, all of the hardening curves had the normal concave-down behavior observed for cubic and non-twinning metals. Thus, reverse testing suggests that the deformation mechanism transition temperature lies between 125 and 150 °C.

A simple method that uses the curvature of the stress–strain curves to determine the twinning–slip transition temperature was adopted based on purely mechanical observations [18,64]. At the transition temperature, the deformation mechanisms change from twinning and slip (low temperature) to slip only (higher temperature). The curvature of the compression stress–strain curves can be used as an indicator to distinguish between twinning-dominant and slip-dominant deformation modes. The sign of the curvature of the compressive stress–strain curve changes from positive for the twinning-dominant to negative for the slip-dominant deformation modes.

**Figure 19.**
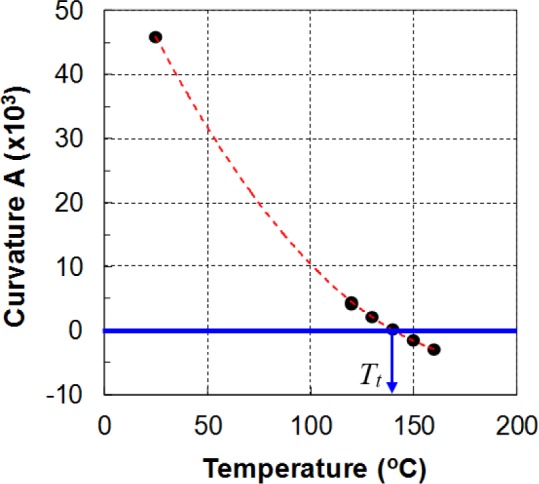
Determination of the twin-slip transition temperature.

The procedure to identify the transition point is briefly presented here. Interested readers may refer to the work of Piao [18] for a more detailed description. First, the data of the stress–strain curves from the monotonic compression tests are fitted using a quadratic equation, $\sigma =A\varepsilon^{2}+B\varepsilon +C$, for an effective strain range, say 0.03–0.08. Second, the curvatures of these fitted curves determined by $A=d^{2}\sigma /d\varepsilon^{2}$ are plotted *versus* temperature. Finally, a curvature–temperature (*A–T*) plot is used to determine the transition temperature, as shown in Figure 19. The *A–T* data are approximated by a smooth curve to find the intersection with the *A* = 0 line. This point defines the transition temperature, as indicated by *T_t_* in Figure 19. For the investigated 1-mm-thick POSCO AZ31B alloy sheets, the calculated value of the twinning–slip transition temperature was approximately 140 °C.

#### 3.2.3. Hardening Model

The existing hardening models (Armstrong–Frederick [65], Chaboche [66], and Yoshida–Uemori [44] models) are suitable for metals that have a stress–strain relationship in the form of an exponential function. For Mg alloys, the hardening behavior is unusual: the stress–strain curves exhibit a sigmoidal shape containing inflection points when the material sheet is in compression or in tension following compression. Therefore, the conventional Chaboche hardening equations fail to fit the experimental data. Several alternatives have been proposed, e.g., the two-surface model [67]. The authors of this paper have also proposed an effective hardening rule that could reproduce the unusual stress–strain curves of Mg alloy sheets during tension–compression cyclic loading testing [63]. A brief description of this model is given below.

The hardening response for deformation modes is characterized using the one-dimensional cyclic loading test data as follows:

$${\bar{\sigma }}_{iso}({\bar{\varepsilon }}_{□}^{p})=R_{□}+\sigma_{0,□}=\sigma^{rev.}-\alpha_{□}$$ (2)

$$R_{□}=\frac{a_{□}}{1+d_{□}\text{exp}\left(-\frac{{\bar{\varepsilon }}_{□}^{p}-{\bar{\varepsilon }}_{□,0}^{*}}{e_{□}}\right)}+Q_{□}\left(1-e_{□}^{-b_{□}({\bar{\varepsilon }}_{□}^{p}-{\bar{\varepsilon }}_{□,1}^{*})}\right)$$ (3)

$$\alpha_{□}=\frac{C_{□}}{\gamma_{□}}\left(1-e^{-}{}^{\gamma_{□}{\bar{\varepsilon }}_{□}^{p}}\right)$$ (4)

where the subscript □ indicates slip (S), twinning (T) and untwinning (U) deformation modes, respectively; σ^*rev.*^ and σ_□_ are reverse loading yield stress and back-stress, respectively. The constants to be determined from the tests after curve-fitting are *a*, *d*, *e*, *Q*, *b*, *c*, *γ*, ${\bar{\varepsilon }}_{T,0}^{*}$, ${\bar{\varepsilon }}_{T,1}^{*}$, ${\bar{\varepsilon }}_{U,0}^{*}$, ${\bar{\varepsilon }}_{U,1}^{*}$ and σ_0_. These parameters can be functions of the pre-strain, and they have the same mathematical meaning in the twinning and untwinning rules. Note that the first term on the right side of Equation (3) defines a sigmoidal function, taking into account the contribution of the unusual twinning/untwinning behavior for the twinning-dominant regime. The term vanishes at the twinning–slip transition temperature, and above it for the slip-dominant regime. The second term represents the physical phenomenon (experimental observation) that a slip mode occurs upon exhaustion of twinning/untwinning [45] at low temperature. The slip hardening rate after all twinning has been nucleated (if the material has not yet failed) is expected to be lower than that of the slip deformation mode itself due to the twinning boundaries acting as a barrier to dislocation motion [50].

**Figure 20.**
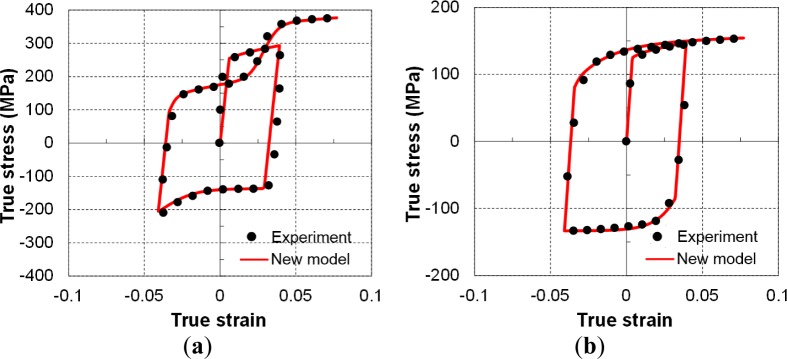
Verification of the proposed hardening model using cyclic test data at: (**a**) room temperature and (**b**) 150 °C.

The general hardening model for plane stress state extended from the above discussion was implemented in a user-defined material subroutine (UMAT) in ABAQUS [53] and PAM-STAMP [63] to enhance the prediction capability of the abnormal hardening features. In which, a reverse loading criterion was introduced to track the change in loading direction of a general stress state and thus the model was capable to describe general non-monotonic loading cases. Figure 20 shows the validation of the TCT flow curves with prescribed pre-strains. The calculated stress–strain curves from the new model matched the experimentally measured flow curves well. More details on calibration and verification of the hardening model are provided in [63].

## 4. Conclusions

The mechanical behavior of AZ31B Mg alloy sheets was characterized experimentally under both monotonic and cyclic loadings. Besides the temperature dependent characteristics of the flow stress, anisotropy and ductility, the transition temperature between the twinning-dominant and slip-dominant deformation mechanisms of strain was also determined from the monotonic tensile tests. In addition, the unloading–loading tensile test results revealed that different laws should be used for the evolution of Young’s modulus in tension and compression loading. A newly developed testing system was successfully used to obtain the reversed stress–strain curves. The developed system proved to be effective and accurate for measuring the stress–strain curves for all of the investigated cases of loading reversal. Tension–compression flow curves were obtained at different temperatures, along with the cyclic behavior of the AZ31B Mg sheets. The results indicated the nature of the reversed-loading behavior, such as a considerable Bauschinger effect, transient behavior, and variable permanent softening responses. The temperature-dependent characteristics of AZ31B Mg sheets were investigated extensively. Accordingly, the hardening behavior was unique below a critical temperature, and a different amount of asymmetry under cyclic loading at different temperatures was observed. Below the transition temperature, the stress–strain curves exhibited unusual behavior: they were concave-up in compression loading and S-shaped in tension following compression loading. The normal form of the flow curve was obtained above the transition temperature. Finally, the proposed hardening model successfully reproduced the cyclic stress–strain curves, including yield asymmetry, a Bauschinger effect, transient and permanent softening, and the unusual concave-up shape.

## Figures and Tables

**Table 1.** Chemical composition of AZ31B Mg alloy sheets (wt%).

Elements	Al	Zn	Ca	Mn	Fe	Cu	Mg
**Composition (wt%)**	2.7~3.1	0.7~0.9	0.002	0.2~0.4	0.005	0.003	balance

**Table 2.** Temperature-dependent Young’s modulus and the indicator of springback amount.

Temperature		RT	100 °C	150 °C	200 °C	250 °C
Young’s modulus (GPa)	43.050	38.121	32.187	29.810	28.952
	$\sigma_{Y}^{T}$	197.112	138.862	103.713	62.673	40.097
Yield stress (MPa)	$\sigma_{Y}^{C}$	122.967	117.544	102.566	55.214	40.223
	σYTE	0.00458	0.00364	0.00322	0.0021	0.00138
Ratio	σYCE	0.00286	0.00308	0.00319	0.00185	0.00139
